# Modeling land use and land cover dynamics of Bale Mountains National Park using Google Earth Engine and cellular automata–artificial neural network (CA-ANN) model

**DOI:** 10.1371/journal.pone.0320428

**Published:** 2025-04-30

**Authors:** Firdissa Sadeta Tiye, Diriba Korecha, Tariku Mekonnen Gutema, Dessalegn Obsi Gemeda

**Affiliations:** 1 Department of Natural Resource Management, College of Agriculture and Veterinary Medicine, Jimma University, Jimma, Ethiopia; 2 California University Santa Barbara, Climate Hazards Center, Famine Early Warning Systems Network Ethiopia Office, Addis Ababa, Ethiopia; Wallaga University, ETHIOPIA

## Abstract

This research aimed to assess the observed land use and land cover (LULC) changes of Bale Mountains National Park (BMNP) from 1993 to 2023 and its future projections for the years (2033 and 2053). The study utilized multi-date Landsat imagery from 1993, 2003, 2013, and 2023, leveraging Landsat 5 TM, Landsat 7 ETM+, and Landsat 8 OLI-TIRS sensors for LULC classification. Standard image pre-processing techniques were applied, and composite images were created using yearly median values in Google Earth Engine (GEE). In addition to satellite data, both physical and socioeconomic variables were used as input for future LULC modeling. The Random Forest (RF) classification algorithm was used for image classification, while the Cellular Automata Artificial Neural Networks (CA-ANN) model within the Modules for Land Use Change Simulations (MOLUSCE) plugin of QGIS was employed for future LULC projection. The analysis revealed significant LULC changes in BMNP, from 1993 to 2023, primarily due to anthropogenic activities, with further changes anticipated between 2023 and 2053.The results showed a notable increase in woodland and shrubs at the expense of grassland and Erica forest. While woodland and shrubs increased by 87.18% and 36.7%, areas of Erica forest and grassland lost about 25% and 22% of their area, respectively, during this period. The LULC model results also indicated that areas covered by woodland and shrubs are expected to increase by 15.97% and 15.57%, respectively, between 2023 and 2053. Conversely, land areas occupied by cultivated land, Erica forest, grassland, and herbaceous plants are projected to decrease by 28.52%, 3.28%, 19.03%, and 6.55%, respectively. Proximity to roads and urban areas combined with rising temperatures and altered precipitation patterns emerged as critical factors influencing land use conversion patterns in BMNP. These findings underscore the complex interplay between environmental factors and human activities in shaping land cover dynamics. Hence, promoting sustainable land management practices among the park administration and local community as well as enhancing habitat protection efforts are recommended. Additionally, integrating advanced remote sensing technologies with ground truthing efforts will be essential for accurate assessments of LULC dynamics in this critical area of biodiversity.

## 1. Introduction

Land use and land cover (LULC) changes are major environmental concerns, which are mostly driven by a multitude of factors, including population growth, industrialization, urbanization, and agricultural expansion. These changes have widespread impacts affecting biodiversity, ecosystem functions, and climate regulation both at global and local scales [1]. Over the past century, LULC changes have accelerated, with forests being converted into agricultural lands, wetlands being drained for development, and natural landscapes fragmented by urbanization [2,3].

Globally, changes in land use and land cover (LULC) are significant contributors to deforestation, the decline of biodiversity, and the rise in greenhouse gas emissions [4] reports that approximately 178 million hectares of forest—around 3% of the global forest area—were lost between 1990 and 2020 [5]. This deforestation was largely driven by the expansion of agriculture, logging activities, and infrastructure development, resulting in habitat destruction, soil erosion, and alterations to water cycles. Such changes not only pose a threat to global biodiversity but also compromise essential ecosystem services that forests provide, including carbon sequestration and water management [6–9].

Africa has also undergone significant changes in LULC due to a combination of population growth, agricultural expansion, deforestation, and urbanization [10]. Reports indicate that the continent loses approximately 3.9 million hectares of forest each year, while the area dedicated to agriculture in Africa has increased by about 35% since the early 2000s [11]. Rapid urban growth in African cities has led to significant changes in land use. The urban population in Africa is expected to double by 2050, resulting in the expansion of urban areas at the expense of agricultural and natural lands [12]. The LULC change in Africa has impacts on deforestation, which significantly threatens biodiversity. The conversion of forests to agricultural land, urbanization, and other forms of land-use intensification have significantly impacted both plant and animal species, causing reductions in their populations and distribution [13]. LULC changes in Africa have posed significant threats to endemic species, many of which are found in isolated ecosystems such as mountains, forests, and islands. Recent studies show that populations of the Mount Kenya elephant shrew have declined by approximately 40% in the last decade due to degradation of their habitats for agricultural expansion, illegal logging, and land encroachment [14–15].

LULC also severely affects hydrological systems in the African continent. For instance, wetlands in Africa have experienced significant losses and degradation due to LULC, such as agricultural expansion, urbanization, and deforestation. Lake Chad, once one of Africa’s largest freshwater bodies, has drastically shrunk due to irrigation, deforestation, and climate variability. The lake’s surface area decreased from approximately 25,000 km² in the 1960s to less than 1,500 km² in recent years, representing a loss of over 90% of its original size. This has severely impacted wetland ecosystems and the livelihoods of millions who depend on the lake [16].

Ethiopia, like many other developing nations, has been experiencing significant LULC over the past few decades, driven by various factors such as population growth, agricultural expansion, deforestation, and urbanization [6,8,17,18]. A recent report by the World Bank indicates that the country has lost approximately 140,000 hectares of forest annually [19]. Contrary to this, agricultural land has increased from about 11 million hectares in the early 2000s to over 16 million hectares in the recent years [20]. LULC changes are critical factors influencing ecological dynamics, biodiversity, local climate, and human livelihoods [13]. The massive deforestation process in the country has resulted in significant habitat loss, leading to a population decline in species that rely on forest ecosystems. Forest loss and habitat fragmentation in the Bale Mountains for instance have led to a reduction in the population size of Bale monkeys by up to 40% in the last few decades [21].

Ethiopia is also expected to experience significant LULC changes in the coming decades, driven by population growth, urbanization, agricultural expansion, infrastructure development, and climate change. These changes are expected to have far-reaching implications for the country’s biodiversity, water resources, and overall ecological balance [22,23]. Protected areas in Ethiopia, including the Bale Mountains National Park (BMNP), have experienced significant LULC changes in recent years, primarily due to human activities such as agricultural expansion, deforestation, and settlement growth. These changes have led to biodiversity loss and ecosystem degradation [24]. The Bale Mountains National Park (BMNP) in the southern highlands of Ethiopia is a notable example of a protected area facing significant LULC pressures [18,25,26]. The park is renowned for its unique biodiversity, hosting several endemic species such as the Ethiopian wolf, the mountain Nyala, and the Bale monkey. Its varied ecosystems, from afro-alpine moorlands to tropical forests, offer critical services, including carbon storage, water regulation, and wildlife habitats. The area was recognized as one of the world’s greatest biodiversity hotspots and enjoys international recognition in conservation management and hence registered as a world heritage center by UNESCO in 2023. However, recent population growth and rising demand for agricultural and grassland have escalated the pressure on the park’s ecosystems [24,26,27].The area is increasingly under threat from human activities, particularly agricultural expansion and the encroachment of settlements [6,28]. The rapid expansion of settlement in and around the park [28] expansion of agricultural lands at the expense of forest land [29] (overgrazing [30] and frequent occurrence of forest fires in 1971, 1973, 1984, 1991, 1992, 1993–1994, 200 and 2007 in the area [31] were among the major threats affecting LULC dynamics of the area. These activities not only threaten the park’s biodiversity but also jeopardize its role as a key water source for millions of people in the region. Though numerous studies have been conducted in this area, they have all emphasized historical land use and land cover (LULC) changes and their major causes, primarily using conventional methods of LULC classification that offer limited classification accuracy. These methods face challenges when dealing with large-scale datasets or high-resolution images due to computational constraints [32]. This research therefore aims to fill these gaps by employing the random forest (RF) algorism in Google Earth Engine (GEE) for historical LULC classification and a cellular automata–artificial neural network (CA-ANN) for modeling future land use/land cover changes in the area.

The research will provide a detailed understanding of how the park’s land cover has changed over time and forecast potential future changes under different scenarios and the implication of these changes on biodiversity in general and Ethiopian wolves in particular. The findings of the study will provide key insights into land cover transformation, offering useful information for policy makers and conservationists to create suitable strategies aiming at preserving BMNP’s biodiversity and ensuring the continued provision of vital ecosystem service for future generations.

## 2. Materials and methods

### 2.1. Description of the study area

#### 2.1.1. Location.

**Fig 1:**
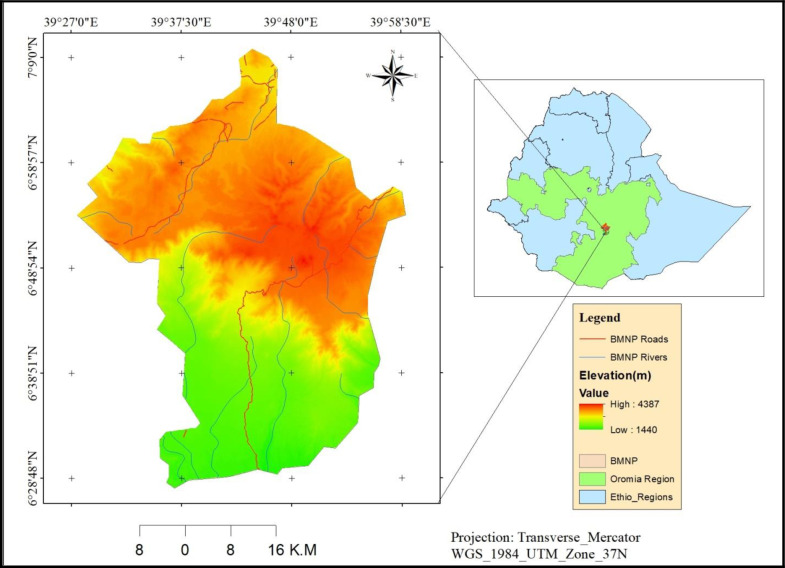
Location map of the study area.

This study was conducted in BMNP in Bale Zone of Oromia National Regional State, Southern Highlands of Ethiopia (**Fig 1**). It lies between 6º30’-7º00’N and 39º30’-39º55’E, 400km southeast of Addis Ababa, the National Capital. Temperatures in BMNP vary significantly, ranging from a minimum of −3ºC over the top of the mountains to a maximum of 25ºC at the lowest elevation [31](). The region experiences bimodal rainfall, with heavy precipitation from July to October and lighter rains from March to June. Mean annual rainfall in the area ranges from 1,000–1,400 mm. BMNP is noted for its diverse biophysical characteristics; the eastern part is dominated by highlands that rise to 3,200 m, while the western lowlands have an elevation of 1,175 m at their outlet [33,34].

#### 2.1.2. Geology and soils.

The Bale Mountains National Park (BMNP) showcases diverse geology and soils, essential to its ecosystems. The geological history of the park dates back to the Miocene and Oligocene periods (approximately 38–25 million years ago), during which extensive volcanic activities formed a large lava plateau. This volcanic activity created cones, such as the Sanetti Plateau, exceeding 4200 meters in elevation. They became separated from the western Ethiopian highlands with the formation of the Rift Valley and, more recently, were divided from the Arsi Mountains due to erosive processes in the upper Wabe Shebelle basin [34]. Glacial activities during the Pleistocene further shaped the landscape, leaving behind distinct glacial valleys and morains.These processes, combined with tectonic uplift, have resulted in BMNP’s characteristic highland and escarpment features [27,35,36]

Soils in the BMNP tend to be shallow, gravelly, and recently derived from volcanic rock exposed since glacial retreat [27]. The soils of the area are closely tied to its geology, topography, and vegetation. At higher altitudes, shallow, organic-rich Andosols dominate, supporting Afro-alphine flora adapted to extreme conditions [37]. Lower altitudes such as the Harenna Forest, contain deeper Nitisols,which are highly fertile due to their volcanic origin [38].However, anthropogenic activities, including deforestation, land use changes, and quarrying, have contributed to soil degradation,erosion,and.However, anthropogenic activities, including deforestation, land use changes, and reduced fertility in some areas [39]. Recent studies reveal that, agro-climatic variations and changing land use patterns have significantly impacted the physical and chemical properties of soils in BMNP. These changes have led to decreased soil fertility and biodiversity, particularly in zones experiencing intensive agricultural pressure and deforestation [38,39].

#### 2.1.3. Land use and land cover (LULC).

The Bale Mountains National Park (BMNP) exhibits diverse land use and cover types shaped by variation in altitude, topography, and ecology. The montane forests (1,500–3,000 m) dominate the western and southern regions, while grasslands and Afro-alpine vegetation prevail above 3,000 m, particularly on the Sanetti Plateau, home to the Ethiopian wolf [35,40]. Cultivated and grazing lands are common at 1,500–2,500 m near park boundaries. Rocky and shallow soils on slopes above 3,000 m support sparse vegetation, while degraded areas near boundaries show deforestation and erosion. Above 3,500 m, rocky, bare zones like the Sanetti Plateau exhibit rugged terrain and minimal vegetation [27,29,40,41].

### 2.2. Data acquisition and pre-processing

Landsat multispectral images are the most widely used for time series analysis of LULC classification due to the long historical data available in their archive [29,42]. In this study, we used multi-date Landsat imagery for the years 1993, 2003, 2013, and 2023 acquired by Landsat 5 Thematic Mapper™, Landsat 7 Enhanced Thematic Mapper plus (ETM+),and Landsat 8 Operational Land Imager (OLI) sensors from the freely available data catalog in GEE at a spatial resolution of 30m in the World Geodetic System (WGS84) (https://earthexplorer.usgs.gov/).Standard image pre-processing, including cloud filtering, topographic, atmospheric, and geometric corrections, layer stacking, and re-sizing, were performed in GEE. A yearly (from 1st January to 31st December) median value was used to create a composite image for the selected years. In addition to this, physical variables, such as DEM data and distance from the streams, as well as socioeconomic factors, such as road networks, towns/settlements population data, and rainfall and temperature data were collected from Ethiopian Meteorological Institute (EMI) and used for future LULC prediction.

DEM data was obtained from the Shuttle Radar Topography Mission Digital Elevation Model (SRTM DEM at 30 m resolution) (https://earthexplorer.usgs.gov/) and used to drive topographic attributes such as slope, aspect, and elevation in ArcGIS 10.8 environment. The road network was obtained from the Planning and Economic Development Bureau of the Oromia region, and the stream network was obtained from the open street map of Ethiopia for the year 2023 (https://extract.bbbike.org). Proximity factors such as distance from roads, rivers, and town/settlement centers were calculated using Euclidean distance in Arc GIS 10.8 (Table 1). Finally, they were clipped to the study area and projected to WGS_1984_UTM_Zone_37N. Thirty years (1993–2023) of daily precipitation and temperature (maximum and minimum) data of 15 meteorological stations found in and around BMNP, taken from the Ethiopian Meteorological Institute (EMI), were organized in Excel, imported into Arc GIS, corrected for their geometry, and then interpolated using the IDW method(Table 1).

**Table 1:** Data types and their sources.

Category	Description	Source
Multitemporal Data	LULC 1993, 2003, 2013 and 2023	Classified from Landsat images
Socioeconomic Factors	Distance from roads	Calculated from the road network
	Distance from towns	Calculated from town data
	Population density	Calculated from the total population
Physical Factors	Distance from streams	Calculated from stream network
	DEM	https://earthexplorer.usgs.gov/)
	Slope	Calculated from DEM
Climate Factors	Precipitation Temperature	Ethiopian National Methodology Institute

### 2.3. LULC classification

There are several advanced non-parametric machine learning classification methods available in GEE for supervised image classification, including Random Forest (RF), Support Vector Machines (SVM), and classification and regression trees, among others [43–45]. For this study, the RF classification algorithm was chosen to identify and differentiate LULC classes in Bale Mountains National Park due to its superior classification accuracy and reliability compared to other machine-learning [44,46–49]). RF is a user-friendly algorithm that only requires the adjustment of two parameters for optimization and can manage large datasets, noisy data, and outliers while minimizing over fitting. The algorithm can also estimate missing values by calculating the proximity between samples [50]. Based on the classification results, seven land use classes were identified: natural forest, woodland, Erica Forest, cultivated land, grassland, herbaceous plants, and shrubs land (Table 2).

**Table 2:** Definition of LULC classes.

LULC Type	Description
**Natural Forest**	Areas that are covered with dense evergreen natural forest with closed canopies.
**woodlands**	The land is covered with both open and closed (high) woodland with dominant species of Acacia-Commiphora vegetation. It also includes scattered Woodland trees
**Grassland**	Extensive grasslands are dominated by grasses with sparse trees and shrubs along the Afromontane range.
**Cultivated land**	Made to include areas allotted to rain-fed cereal crops (e.g., Corn, Barley, Teff, and Wheat.
**Erica forest**	It is an area of ericaceous belt comprised of forest, thickets, and scrublands of E.trimera and Erica arboreal communities.
**Shrubs land**	area with trees that are not evergreen during the dry season
**Herbaceous plants**	Located above the forest, afro-alpine vegetation includes dwarf shrubs, helichrysum splendid, Alchemilla human, and the giant lobelia.

To compare changes in LULC area along elevation gradients, the study area was classified into five elevation zones (<2000meters, 2000–2600 meters, 2600–3200 meters, and > 3200 meters) using DEM and Arc GIS tools. Finally, areas for each land use class were extracted with the help of a tabulated area tool in Arc GIS software.

### 2.4. Accuracy assessment

Accuracy assessment validates the classification by assessing how pixels are assigned to the correct LULC classes. In this study, Google Earth’s historical satellite image and ground truth data were used to assess classification accuracy. Based on the recommendation of scholars [45,51,52], around 60 ground-based training samples were generated for each LULC class to construct LULC maps. The accuracy of the classified maps was determined by superimposing 400 Global Positioning System (GPS) and 350 Google Earth image random sample ground truth data on top of them. The user accuracy (Eq. 1), producer accuracy (Eq. 2), and overall accuracy (Eq. 3) and kappa statistics (Eq. 4) were calculated for the accuracy assessment using the following formulas:


$$UsersAccuracy=\frac{TotalNumberofcorrectlyclassifiedpixels}{Sumofrawsinthatcategory}*100$$ Eq. 1

$$ProducersAccuracy=\frac{TotalNumberofcorrectlyclassifiedpixels}{Sumofcolumninthatcategory}*100$$ Eq. 2

$$Overallaccuracy=\frac{TotalNumberofcorrectlyclassifiedpixels}{Totalnumberofreferencepixels}*100$$ Eq. 3

$$Kappacoefficient\left(K\right)=\frac{\left(TS*TCS\right)-\sum \left(Columntotal*Rawtotal\right)}{TS^{2}-\sum \left(Columntotal*Rawtotal\right)}*100$$ Eq. 4

Where; TS = Total sample and TSS = Total number of correctly classified pixels.

### 2.5. LULC change analysis and transition potential modeling

The LULC change analysis shows the spatiotemporal variations as well as the gains and losses of areas of LULC classes of the same area at different periods. It is an important tool to generate evidence for decision-makers, spatial planners, local communities, or actors who are operating within a given landscape to formulate appropriate policies and strategies, create data for spatial planning, and develop detailed land use plans as well as understand agents of change [29].

This study utilized land use maps 1993, 2003, 2013, and 2023 to analyze changes in LULC across four distinct periods: 1993–2003, 2003–2013, 2013–2023, and the overall span from 1993 to 2023. To assess the spatiotemporal changes and LULC transitions during these intervals, the Modules for Land-Use Change Simulation (MOLUSCE) plugin in QGIS, along with ArcGIS tools, was employed. Additionally, the detection of LULC changes was quantified using the percentage and rate of change, as outlined in Eq. 5 and 6.


$$Percentageofchange=\frac{A2-A1}{A1}*100$$ Eq. 5

where: $A_{2}$ = Area of LULC in year 2

$A_{1}$ = Area of LULC in year 1


$$Rateofchange=\frac{A_{2}-A_{1}}{N}$$ Eq. 6

where, $A_{2}$ = Area of LULC in year 2


$$A_{1}\;\text{= Area of LULC in year 1}$$

N = Number of years elapsed between $A_{1}$ and $A_{2}$

### 2.6. Preparation and selection of spatial variables for future LULC simulation

**Fig 2:**
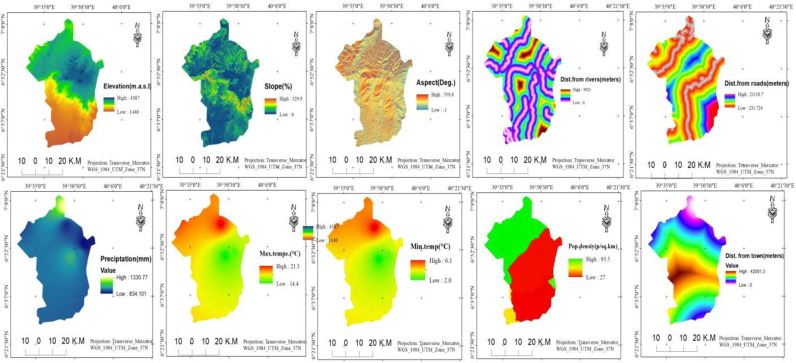
Spatial variables selected for LULC modeling.

To simulate future LULC, it is recommended that several predictor variables, which play a major role in LULC change and transition, have to be taken into account. Based on LULC change drivers reported in previous studies [10,51,53,54], and the availability of such factor datasets, we selected 10 predictor variables to describe the LULC change processes that occurred in BMNP between 1993 and 2023. These include topographic variables such as slope, aspect, elevation, distance from rivers/streams, and human disturbance variables like distance from towns and roads, as well as population density and climate factors (precipitation, maximum temperature, and minimum temperature) (Fig 2). These variables are frequently used to predict LULC because they provide reproducible data on the natural and human disturbances in LULC processes [50].

After selection, geometric matching of the variables, which includes cell size, extent, dimension, and coordinate reference system, was conducted. Accordingly, the cell size and coordinate reference system for all raster variables used in this study are 30m resolution and WGS_1984_UTM_Zone_37 respectively.

The MOLUSCE plugin in QGIS provides several established methods, such as Pearson’s correlation and Cramer’s coefficient, for evaluating the relationship between LULC data and geographic factors [55]. This study used Pearson’s correlation to assess the relationship between the variables since the spatial variables used were not categorical. Pearson’s correlation, denoted as r, is a parametric measure of the linear correlation between two variables. It is defined as the covariance of the two variables divided by the product of their standard deviations, as shown in Eq. 7.


$$r=\frac{\sum_{i=1}^{n}(x_{i}-\bar{x})\left(y_{I}-\bar{y}\right)}{\sqrt{\sum_{i=1}^{n}(x_{i}-{\left(\bar{x}\right)}^{2}\sum_{i=1}^{n}(y_{I}-{\left(\bar{y}\right)}^{2}}}$$ Eq. 7

Where $\bar{x}and$$\bar{y}$ are the sample mean of X_1_, X_2_... X_n_ and Y_1_, Y_2_... Y_n_, respectively.

Pearson’s correlation coefficient, r, ranges from −1–1. A value of 0 indicates no linear relationship between the two variables, while values of 1 and -1 signify perfect positive and negative correlations, respectively.

### 2.7. Future LULC Prediction and Validation

After generating LULC maps for the years 1993, 2003, 2013 and 2023, future LULC predictions were performed for the years 2033 and 2053 using the CA-ANN algorithm in the MOLUSCE plugin embedded in QGIS software version 2.18.15. Studies have shown that the CA-ANN model is more powerful and robust in simulating future LULC as compared to other models, such as linear regression and Marcov [50]. Moreover, the MOLUSCE plugin effectively processes LULC change analysis and is suitable for evaluating spatiotemporal LULC changes and predicting future scenarios([45,46,56,57]. For future LULC predictions, we retained the same resolution (30*30 m) and WGS84 coordinate system.

**Fig 3:**
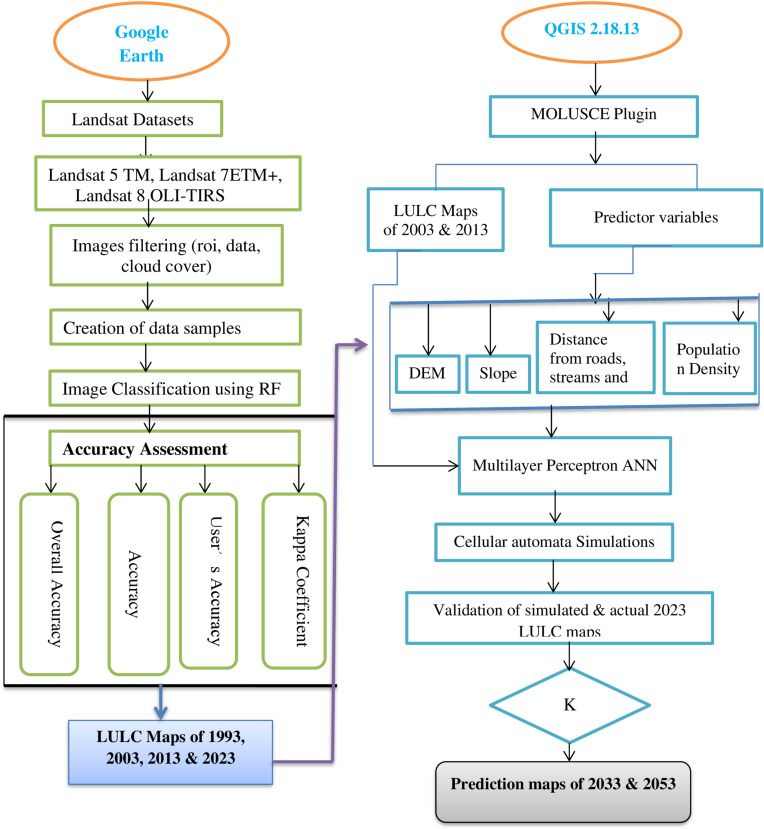
General methodological flow chart of LULC change and its prediction.

Prediction of potential LULC for a prospective project can only be reliable if the simulation outcome is validated using existing datasets (Fig 3). Accordingly, we first simulated LULC for 2023 using LULC maps of 2003 and 2013 and the selected predictor variables. After that, the validation process was performed using a comparative analytical procedure of the overall correctness percentage and kappa coefficient in the MOLUSCE plugin. Specifically, to validate the performance of the CA-ANN model, we compared the simulated LULC map for 2023 that was generated using the CA-ANN algorithm with the one generated for the same year using the multi-date Landsat images and RF classifier. After obtaining adequate validation metrics, we utilized LULC data from 2013 and 2023 to simulate future LULC in 2033 and 2053 So,.

## 3. Results and discussion

### 3.1. Land use land cover change

**Fig 4:**
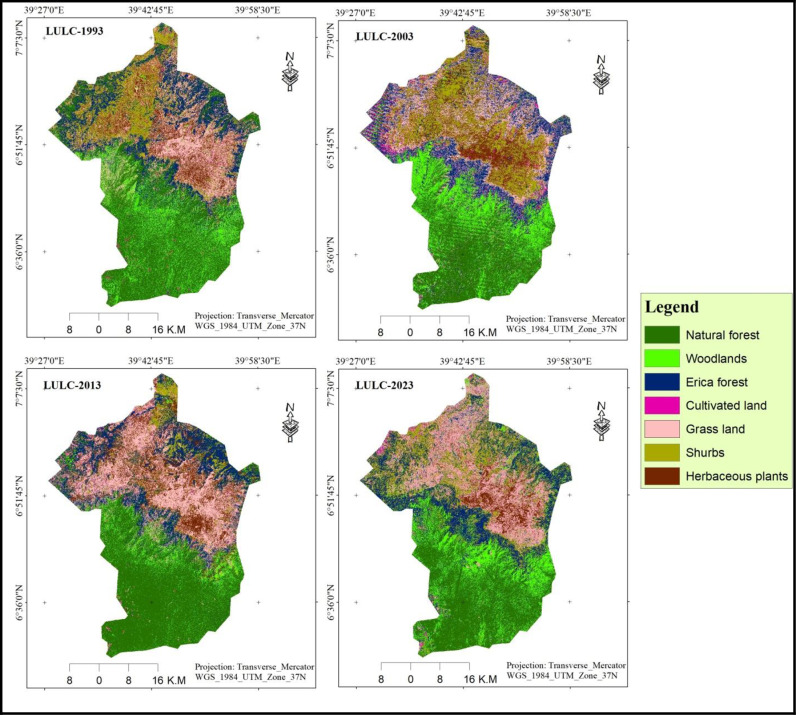
LULC maps of BMNP for the years 1993, 2003, 2013 and 2023.

To assess changes in land use within BMNP, LULC classes for 1993, 2003, 2013, and 2023 were identified using the RF classification algorithm in Google Earth Engine and analyzed with the help of ArcGIS (Fig 4 and Table 3) The results clearly show that the majority of the BMNP landscape was covered by natural forest (31.24%), followed by grassland (21.62%), Erica Forest (16.34%), and shrub land (10.6%) in 1993. Together, these land use classes accounted for approximately 80% of the total area of BMNP. In contrast, woodlands, herbaceous plants, and cultivated land comprised only about 20% of the area, covering approximately 9.67%, 9.68%, and 0.83% of the total land area, respectively. A similar trend in LULC changes was observed in 2003, with natural forest representing the largest percentage (32.83%) of the total area, followed by grassland (19.22%), Erica Forest (15.98%), and shrub land (12.39%). These land uses also accounted for around 80% of the total land area of BMNP, while other land use types, including cultivated land, herbaceous plants, and woodlands, occupied the remaining 20%. In 2013, the natural forest remained the dominant LULC type, followed by grassland, shrub land, and Erica Forest, which covered approximately 31.40%, 18.67%, and 12.81% of the park’s land area, respectively. Conversely, woodlands, herbaceous plants, and cultivated land represented the smallest percentages in 2013, covering 10.57%, 9.08%, and 0.49% of the study area, respectively.

**Table 3:** Areas of LULC classes of BMNP in the years (1993, 2003, 2013, and 2023).

LULC-types	1993		2003		2013		2023	
	Area Km^2^	%	Area Km^2^	%	Area Km^2^	%	Area Km^2^	%
Cultivated land	20.14	0.83	23.11	0.95	11.97	0.49	11.78	0.48
Erica forest	397.65	16.34	389.07	15.98	311.82	12.81	297.87	12.24
Grassland	526.31	21.62	467.95	19.22	454.37	18.67	409.41	16.82
Herbaceous plants	235.63	9.68	239.99	9.86	221.1	9.08	205.08	8.43
Natural forest	760.51	31.24	799.21	32.83	764.21	31.40	704.37	28.94
Shrubs land	258.5	10.62	301.55	12.39	413.32	16.98	483.86	19.88
Woodland	235.37	9.67	213.23	8.76	257.32	10.57	321.74	13.22

**Fig 5:**
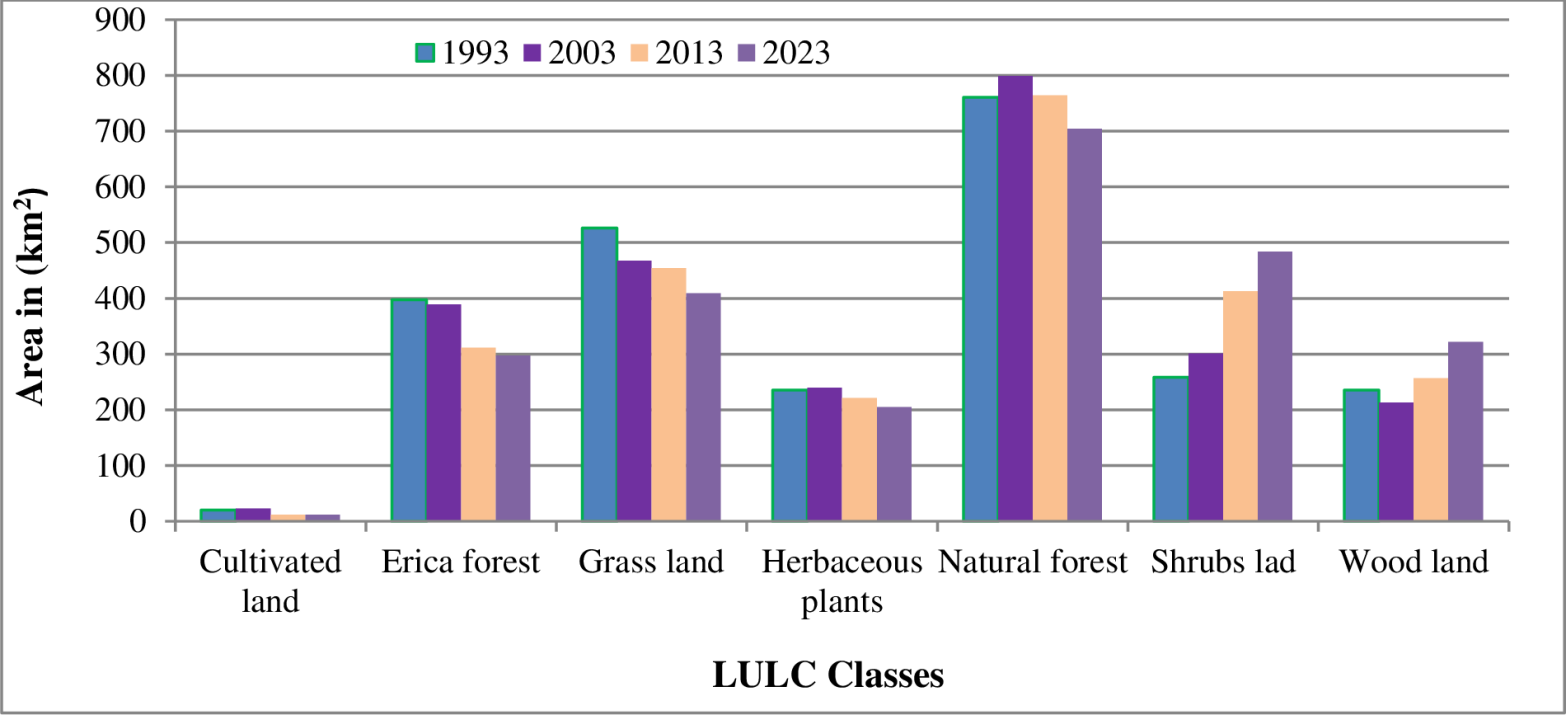
LULC Areas (KM^2^) of BMNP in 1993, 2003, 2013 and 2023.

A similar LULC distribution was observed in 2023, with natural forests ranking first, followed by shrub land, grassland, and woodlands. Together, these land uses accounted for approximately 79% of the park’s total land area. The remaining 21% of BMNP was comprised of Erica Forest (12.24%), herbaceous plants (8.32%), and cultivated land (0.48%). Throughout the study period, natural forests have consistently occupied the largest proportion of BMNP’s total area Table 3 and Fig 5). This reflects the park’s status as a protected area with significant natural habitat remaining intact. However, it is important to note that the extent of dominant land use types has fluctuated over time. The findings of the current study partially align with those of [29] and [58] who also reported the dominance of natural forests in BMNP during their respective study periods. However, their findings indicate a decline in shrub land and an increase in farmland, which contradicts the findings of the current study. This discrepancy may be due to methodological differences, data sources, or the specific periods being compared.

In contrast, these findings agreed with the study by [18], which reported a decline in agricultural land, grassland, and Erica forest within the BMNP between 1973 and 2008 (Fig 4). This study also found a notable decrease in areas of cultivated land in the BMNP in between 1993–2023.This is, potentially due to successful conservation efforts made by the park administration and the local community that aimed at reducing agricultural expansion. This finding contrasts with previous research [18,27–29], who reported an increasing trend in cultivated land within the park. This discrepancy could be explained by variations in the study areas, methodologies, and tools used in each research project, as all employed conventional methods of image classification.

The Ericaceous belt, crucial for water catchment and biodiversity in the BMNP, has experienced a significant decline in the area. This could be due to climate change impacts and forest fires. This is also supported by other research findings. For instance, [59] documented an upward range expansion and an increase in dense Ericaceous vegetation at mid-altitudes, suggesting that climate change is influencing species distribution along elevation gradients. This research reaffirms the critical role of temperature and precipitation conditions in determining the geographic distribution of species.

[60] highlighted the increasing prevalence and severity of forest fires in the BMNP over the past few decades. They reported that, area affected by forest fires increased from 210 hectares to 12,825 hectares in between 1980–2013. Approximately 84% of recent fires incidents were occurred within the BMNP, devastating 60% of the Ericaceous belt, the major water catchment area for the Bale Mountains massif.

### 3.2. Accuracy assessment

Because of its empirical approach, the accuracy of the classified satellite images needs to be assessed. One method is the use of a contingency table (confusion matrix) produced from a random sample of individual pixels or clusters compared to “known” cover conditions over the same pixel areas. Therefore, the confusion matrix method were used to calculate the accuracy of the spectral supervised classification of Landsat ETM, ETM+, and OLI/TIRS images of the study area.

Accordingly, the overall accuracy of the classified images was 95%, 91%, 94%, and 91%, and its kappa (K^) statistics were 92%, 89%, 91%, and 90% for 1993, 2003, 2013, and 2023, respectively (Table 4). The kappa statistics of < 40%, 40–75%, and >70% are considered poor, good, and excellent, respectively [61,62]. Hence, the classified image has a very good agreement with reference information.

**Table 4:** Accuracy assessments of classified LULC classes for the years (1993, 2003, 2013 and 2023).

LULC Classes	1993		2003		2013		2023	
	UA	PA	UA	PA	UA	PA	UA	PA
Cultivated land	83	83	86	86	87	87	83	83
Erica forest	90	82	82	78	86	90	90	93
Grasses	95	97	94	97	93	97	97	91
Herbaceous plants	97	96	94	94	98	97	80	86
Natural forest	93	97	90	91	86	93	100	95
Shrubs	88	88	92	82	83	71	86	90
Woodland	89	85	86	95	96	93	94	94
Overall Accuracy (%)	95		91		94		91	
Kappa Coefficient (%)	92		89		91		90	

### 3.3. LULC changes detection

**Fig 6:**
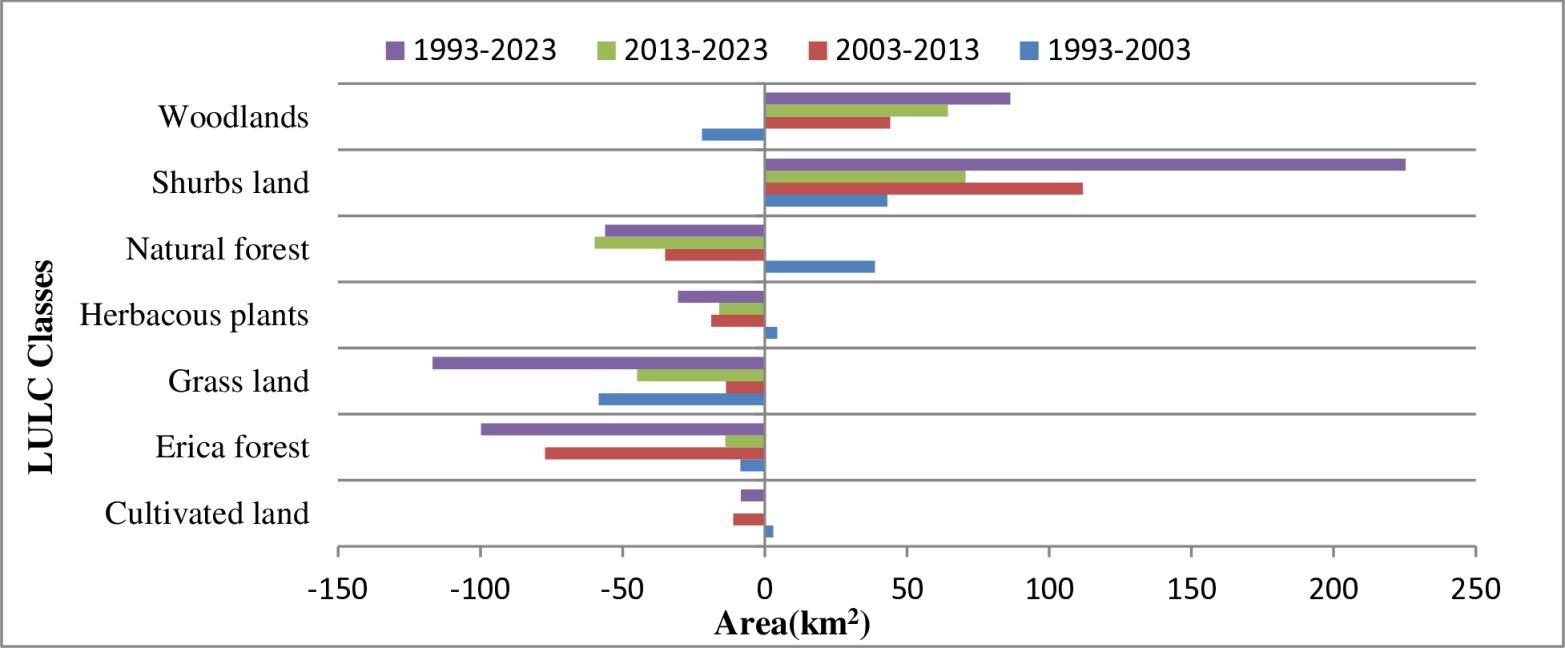
Area of LULC changes in BMNP (1993 – 2023).

LULC change detection analysis of the area shows that the areas of some land use classes increased while the areas of others decreased during the study periods. Land use maps of 1993, 2003, 2013, and 2023 were used to detect changes between each land use class at the decadal level and for the entire study period. The results of the study revealed a notable expansion in areas of shrub land and woodlands from 1993 to 2023 and a shrinking in all other land use classes, mostly cultivated land, Erica Forest, and grassland. Table 5 indicates that the areas of Shrubs land, Natural Forest, Herbaceous plants, and cultivated land were respectively increased by 43.05 Km^2^, 38.70 km, 4.36 Km^2^, and 2.97 Km^2^, whereas cultivated land, woodlands, and shrubs decreased by −58.36 Km^2^, −22.14 Km^2^, and −8.58 Km^2^, respectively in, between 1993 and 2003. Except for shrubs lands and woodland, which were sharply increasing, all other LULC classes were declined in their areas, and hence their annual rate of change during the (2003–2013), (2013–2023), and (1993–2023) periods were negative (Table 5 and Fig 6). This result partially aligns with the findings of [59] however, it contradicts his conclusion, which indicated an increase in cultivated land in the same area.

**Table 5:** Changes in area and the annual rate of growth of LULC classes (km^2^/year) in BMNP (1993 and 2023).

LULC Classes	1993–2003		2003–2013		2013–2023		1993–2023	
	Area (km^2^)	Rate of change (%)	Area (km^2^)	Rate of change (%)	Area (km^2^)	Rate of change (%)	Area (km^2^)	Rate of change (%)
Cultivated land	2.97	1.47	−11.14	−4.82	−0.19	−0.16	−8.36	−12.45
Erica forest	−8.58	−0.22	−77.25	−1.99	−13.95	−0.45	−99.78	−7.53
Grasses	−58.36	−1.11	−13.58	−0.29	−44.96	−0.99	−116.90	−6.66
Herbaceous plants	4.36	0.19	−18.89	−0.79	−16.02	−0.72	−30.55	−3.89
Natural forest	38.70	0.51	−35.00	−0.44	−59.84	−0.78	−56.14	−2.21
Shrubs	43.05	1.67	111.77	3.71	70.54	1.71	225.36	26.15
Woodland	−22.14	−0.94	44.09	2.07	64.42	2.50	86.37	11.01

The reduction in areas of cultivated land was mainly due to the protection of the area from intensive human interference/cultivation. Frequent outbreaks of wildfire, overgrazing, and deforestation were mentioned as the major causes of shrining in the areas of Erica forest, grassland, herbaceous plants, and natural forest during the study period. In addition, seasonal migration of people to the Senate Plateau and informal settlements in different parts of the area were considered contributing factors to LULC dynamics.

### 3.4. LULC transitional matrices

The transition matrix is crucial for examining temporal changes within LULC categories. The rows of the matrix table represent the LULC categories in the first year, while the final year is shown in the columns. Each off-diagonal entry reflects the size of the transition from one class to another, while the diagonal entries display the size of class stability.

The output of the post-classification comparison gives detailed information about the extent and nature of changes that have occurred in each LULC class during the study period.

The results show that, about 38.02%, 45%, 36%, and 55% of the total land use areas of BMNP were converted to other land use types in between 1993–2003, 2003–2013, 2013–2023, and 1993–2023, respectively. Woodland and shrubs were the most unstable LULC classes. About 186.49 (87.46%) and 215.56 (71.48%) of their original areas were converted into other LULC types. The largest share of woodland was converted into natural forest (69.25 km^2^) and shrubs (64.37 km^2^), while (96.49 km^2^) of shrub land was converted into grassland. The major LULC conversions took place in the area in between 1993 and 2023 were shown and summarized in (Tables 6–9).

**Table 6:** LULC transition matrix of Bale Mountains National Park (1993–2003).

LU/LC classes		2003							Total
		Cultivated land	Erica forest	Grassland	Herbaceous plants	Natural forest	Shrubs land	Woodland	
**1993**	Cultivated land	15.20	0.90	1.48	0.46	0.69	0.92	0.49	20.14
	Erica forest	0.34	266.59	31.85	17.91	14.70	45.68	20.58	397.65
	Grassland	4.19	30.21	354.01	11.18	7.17	96.49	23.07	526.31
	Herbaceous plants	0.85	12.80	14.07	176.75	8.63	13.81	8.73	235.63
	Natural forest	0.21	30.48	31.66	12.74	583.49	32.68	69.25	760.51
	Shrubs lad	1.35	30.38	17.62	14.94	43.85	85.99	64.37	258.50
	Woodland	0.96	17.71	17.27	6.02	140.69	25.98	26.74	235.37
	Total	23.11	389.07	467.95	239.99	799.21	301.55	213.23	2434.11

**Table 7:** LULC transition matrix of Bale Mountains National Park (2003–2013).

LU/LC classes		2013							Total
		Cultivated land	Erica forest	Grassland	Herbaceous plants	Natural forest	Shrubs land	Woodland	
**2003**	Cultivated land	6.17	0.61	5.76	4.89	0.45	2.77	2.45	23.11
	Erica forest	0.76	208.90	26.43	14.66	8.84	62.17	67.31	389.07
	Grassland	1.65	23.27	243.74	48.17	46.03	63.79	41.30	467.95
	Herbaceous plants	1.17	22.47	36.86	118.43	7.18	22.17	31.72	239.99
	Natural forest	1.37	21.89	48.25	10.13	600.92	79.24	37.41	799.21
	Shrubs lad	0.32	18.69	44.89	12.28	44.33	134.21	46.83	301.55
	Woodland	0.54	15.99	48.44	12.55	56.46	48.96	30.29	213.23
	Total	11.97	311.82	454.37	221.11	764.21	413.32	257.32	2434.11

**Table 8:** LULC transition matrix of Bale Mountains National Park (2013–2023).

LU/LC classes		2023							Total
		Cultivated land	Erica forest	Grassland	Herbaceous plants	Natural forest	Shrubs land	Woodland	
**2013**	Cultivated land	2.03	1.62	3.90	0.76	0.05	2.38	1.24	11.97
	Erica forest	0.30	181.64	6.77	2.75	3.56	95.91	20.88	311.82
	Grassland	6.77	15.56	269.10	18.45	0.15	111.65	32.68	454.37
	Herbaceous plants	0.26	1.12	73.31	138.05	0.31	7.18	0.87	221.10
	Natural forest	0.40	21.31	8.72	0.16	592.87	27.94	112.82	764.21
	Shrubs lad	1.62	56.20	39.96	44.64	26.69	230.77	13.44	413.32
	Woodland	0.40	20.42	7.65	0.27	80.74	8.03	139.82	257.32
	Total	11.78	297.87	409.41	205.08	704.37	483.86	321.74	2434.11

**Table 9:** LULC transition matrix of Bale Mountains National Park (1993–2023).

LU/LC classes		2023							Total
		Cultivated land	Erica forest	Grassland	Herbaceous plants	Natural forest	Shrubs land	Woodland	
**1993**	Cultivated land	4.99	0.87	3.99	3.22	0.53	3.62	2.92	20.14
	Erica forest	0.46	112.74	29.09	11.35	22.02	176.64	45.36	397.65
	Grassland	1.61	32.86	235.79	50.63	50.99	85.28	69.16	526.31
	Herbaceous plants	0.75	16.99	38.46	131.65	0.11	35.72	11.95	235.63
	Natural forest	1.68	73.30	3.47	1.01	534.04	76.89	70.11	760.51
	Shrubs lad	1.39	38.35	62.71	6.07	20.06	90.75	39.16	258.50
	Wood land	0.90	22.76	35.90	1.16	76.62	14.97	83.06	235.37
	Total	11.78	297.87	409.41	205.08	704.37	483.86	321.73	2434.11

Similarly, from 2003 to 2013, about (88.23%) of woodland and (67.53%) of shrub land were converted into other LULC types. During this period, the largest share of woodland (67.31 km2) was changed into Erica Forest. Natural forests, followed by herbaceous plants, were the most stable LULC types from 1993 to 2023, whereby about 77.58% and 62.44% of their previous area remained the same. Contrary to this, about 82.77% of cultivated land was converted into other LULC classes, mostly grasslands, making it the most unstable LULC type during the same period. An attempt has also been made to analyze LULC changes for the entire study period (1993–2023). The study found that natural forest, which retained 514.04 km² (72.98%) of its original area, was the most stable LULC type. In contrast, shrub land and woodland were the most unstable, losing approximately 81.24% and 73.98% of their original areas, respectively. The largest areas of shrubs land (176.64km^2^) and woodland (99.16km^2^) were respectively converted into Erica Forest and grasslands during this period.

Relatively, areas covered by natural forests were less susceptible to changes than the others, indicating that the vegetation zones are under the management and protection of the Bale Mountains National Park (BMNP) administration. These findings are in agreement with the studies conducted by [29]. Grassland and Erica Forest were the most reduced LULC classes, which lost about -116.90km^2^ and 99.77km^2^ of their area between 1993 and 2023. Overgrazing, resettlement, wildfire, and deforestation were the driving factors. This finding is in line with the findings of other researchers [25,63,64].

### 3.5. LULC changes along elevation gradients

**Fig 7:**
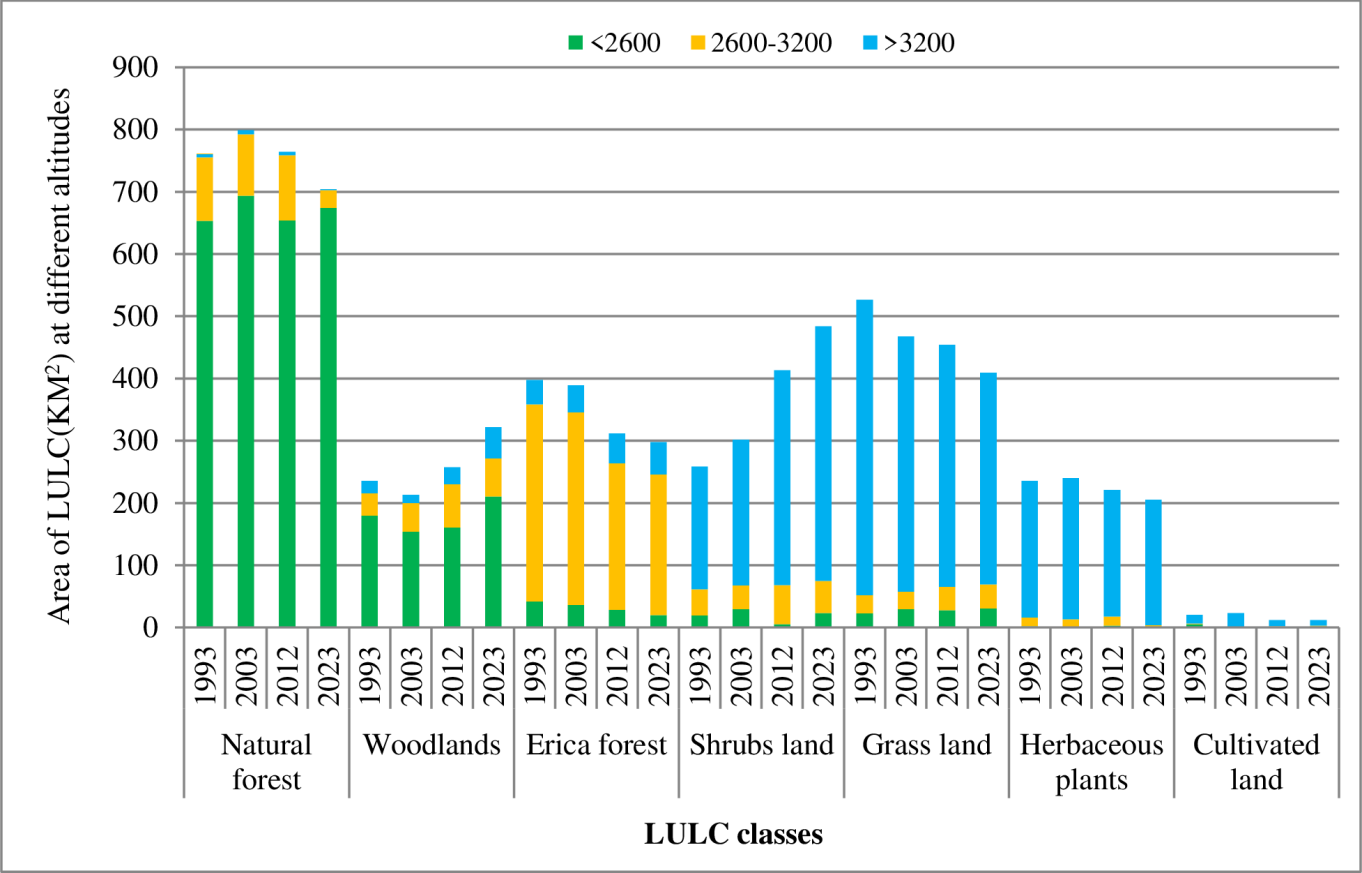
Area of LULC changes in BMNP along elevation gradients (1993 – 2023).

In addition to analyzing the entire LULC changes of BMNP, an attempt was made to examine the nature of LULC changes across different elevation levels using the Digital Elevation Model (DEM) and the Tabulated Area tool in ArcGIS software. The findings of the study reveal significant patterns of LULC change across different altitudes in BMNP over the last three decades (Fig 7 and S1–S2 Tables).

The results indicate that areas of natural forest have increased by 3.2% at lower (< 2,600 meters) altitude; but, decreased by 71.8% and 66.4% at mid (2,600–3,200 meters) and highest (> 3,200 meters) altitudes respectively. The increase in natural forests at lower altitudes (<2600m) is noteworthy, indicating potential forest regeneration or successful conservation efforts in this zone [65]. However, its significant decline at higher altitudes suggests adverse ecological changes. This trend may be attributed to climate change, which can alter temperature and precipitation patterns, affecting the growth and survival of forest species. About these changes, [66] highlights that higher altitudes are typically more sensitive to climate fluctuations, and the decline of natural forests may be related to shifting climatic zones, where species that once thrived at higher elevations can no longer survive due to warming temperatures. Although shrub land and woodlands expanded across all altitudes, their growth was particularly pronounced at the highest altitude (> 3,200 meters), where they increased approximately by 153% and 107.3%, respectively. The observed decline in Erica forests at lower and mid-elevations and their increase at higher elevations suggest that Erica forests are also sensitive to altitude-related climate changes. At lower altitudes, the significant decrease in Erica forests could be linked to land conversion for agriculture, particularly grazing, or changes in vegetation structure due to altered fire regimes. The increase at higher elevations might indicate a shift in suitable habitat due to changing climatic conditions, with species typical of lower altitudes moving upslope in response to warmer temperatures. This upward migration of Erica forests is consistent with global patterns where species are shifting their ranges in response to climate change [18,67,68].

Herbaceous plants play crucial roles in ecosystems as primary producers and as vital components of food webs. Their sharp decline at lower altitudes (with an 81% reduction between 1993 and 2023) was attributed to climate and non-climatic factors. On one hand, lower altitudes are often areas of higher human and livestock density. Overgrazing by livestock can prevent the regeneration of herbaceous vegetation, degrade soil quality, and contribute to erosion. This result coincides with the findings of [69] who highlighted that Grazing pressure is likely to exacerbate the decline in herbaceous cover, particularly in the absence of sustainable land management practices. On the other hand, herbaceous plants are sensitive to changes in temperature, precipitation, and other climatic variables [70–72]. If the climate has become warmer or drier in the lower altitudes, it could have affected the growth and survival of many herbaceous species, especially those adapted to cooler or more temperate conditions [70].Analysis of climate data taken from EMI, shows that, the mean precipitation of the area decreased from 1026 mm to 923 mm while mean maximum and minimum temperatures were respectively increased by 1.5 °C and 1.75 °C from 1993 to 2023. Therefore, elevation shifts of herbaceous plants in BMNP were more experienced due to climate change as it disrupted seasonal growth cycles, which in turn led to a decrease in herbaceous plant populations.

Grasslands exhibited varied trends with a 34% increase at lower altitudes and a 23% increase at mid-altitudes, but a 28.3% decline at the highest elevations. The expansion at lower and mid-altitudes is likely due to the expansion of grazing area or the conversion of forests and woodlands into grasslands for agricultural use, driven by anthropogenic activities. This is also supported by [67] who documented that the expansion of grazing land coupled with agricultural land conversion resulted in increased grassland cover in Bale Mountains at altitudes below 3200 meters. But, the decline of grass lands at the highest elevation could be attributed to temperature changes caused by global warming. These changes may favor a shift toward vegetation types better suited to warmer conditions, such as shrub land or woodlands, as the climate becomes less suitable for grassland species adapted to cooler, high-altitude environments. Additionally, higher altitudes are more sensitive to climate change, and the observed decline may result from reduced precipitation, making grasslands less viable at these elevations. This finding is supported by [73], who studied the response of tropical highland grassland species to climate change in the Arsi mountains of Ethiopia. Their research projected a warming trend that significantly reduced the altitudinal ranges and habitat areas of all grasslands.

The findings of this study reveal significant patterns of LULC change across different altitudes in BMNP over the last three decades. These changes are the result of the combined effects of human activities, climate change, and land management practices. There are considerable research findings supporting the upslope shifts of LULC in mountainous areas as a result of climate warming. Studies in the Rocky Mountains and other ranges in North America have shown high-velocity shifts in land use/land cover, with shrub land and woodlands expanding into higher altitudes [74–76,77] also highlights a significant ecological shit in the Rocky Mountain alpine region, where shrubs cover expanded by 441% over 62years (1946–2008) and continues to grow at an exponential rate. An increase in shrub biomass, cover and abundance have also been observed in many Arctic, high-latitude and alpine tundra ecosystems over the past century. They indicated that, warming temperatures, changes in snow cover, altered disturbance regimes as a result of permafrost thaw, tundra fires, and anthropogenic activities are all contributing to observed changes in shrub abundance. Analysis using Landsat satellite data reveals that these vegetation types are increasing in area and moving to new elevations where they previously did not thrive. These shifts are often driven by rising temperatures and decreasing and changing precipitation patterns, enabling vegetation adapted to warmer conditions to colonize higher elevations. [78] compared the magnitude of observed elevation range shifts of different taxonomic groups with the magnitude of expected range shifts given climate change alone in the French mountain forests. They found that, with increasing temperature by 0.17 1 °C per decade, plant species shifted toward higher elevations on average by 29 m per decade, considering an adiabatic lapse rate of 0.6 °C per 100 m in that region between 1965 and 2005.

Since BMNP is a protected area, the changes in LULC across altitudinal gradients observed on the last three decades are mainly the result of climate change (decreasing precipitation and increasing both maximum and minimum temperature than other factors.

### 3.6. Transition potential modeling and model validation

**Fig 8:**
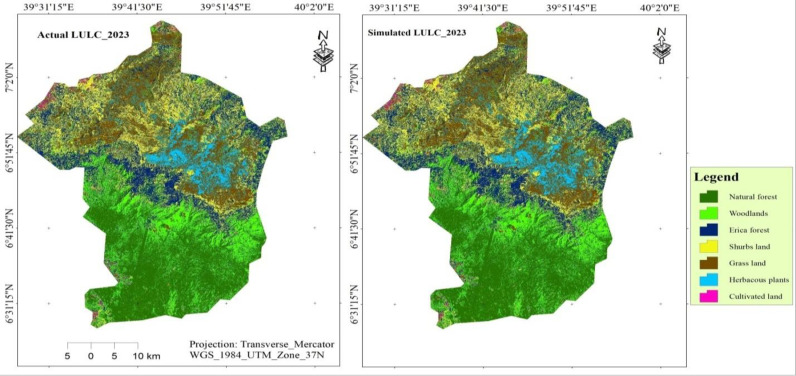
Actual and simulated LULC map of BMNP for the year 2023.

The MOLUSCE plugin integrates some well-known algorithms for transition modeling, such as the ANN (multiplayer perceptron), weights of evidence, multicriteria evaluation, logistic regression, and CA algorithm for future simulation [78,79]. The CA-ANN approach was used for transition potential modeling and prediction. We employed LULC data from 2003–2013 along with spatial variables to project LULC for 2023 and obtained a validation kappa value of 0.97. After obtaining the projected LULC, we compared the actual and projected LULC of 2023 using the MOLUSCE kappa validation technique Fig 8 and Table 10). Thus, we found that the kappa (histogram) was 0.97, the kappa (overall) was 0.86, the kappa (location) was 0.89, and the % of correctness was 88.8%(S1 and S2 Figs). This indicates that the model is performing well in BMNP. Fig 8 and Table 10 show the actual and projected maps and statistics for 2023.

**Table 10:** A reas of actual and simulated LULC in 2023.

LULC Type	Actual LULC (2023)		Simulated LULC (2023)	
	Area(km^2^)	%	Area(km^2^)	%
Cultivated land	11.78	0.48	11.77	0.48
Erica forest	297.87	12.24	293.80	12.07
Grassland	409.41	16.82	402.86	16.55
Herbaceous plants	205.08	8.43	204.22	8.39
Natural forest	704.37	28.94	697.13	28.64
Shrubs land	483.86	19.88	498.79	20.49
Woodland	321.74	13.22	325.55	13.37
**Total**	**2434.11**	**100.00**	**2434.11**	**100.00**

The comparison between both output maps provided significant accuracy (up to 90%). Therefore, it was noteworthy to use the MOLUSCE model for future land use prospects [56].Thus, the land use maps of 2013 and 2023 were used to project for 2033 and similarly, output maps of 2023 and 2033 were used to project for 2053 LULC.

Additionally, accuracy can also be determined by the neural network curves [54,56,80]. These curves can be under-fit, over-fit, and good-fit, reflecting the prediction’s accuracy (correctness). Under-fitting occurs when a model is too simplistic for data, over-fitting occurs when a model is too sophisticated for data, and good fitting occurs when both the validation and training data are symmetrical. Moreover, the neural network learning curve (NNC) also provides the cross-validation of future simulations. Neural network learning curves are commonly employed in machine learning for algorithms like deep learning neural networks [56,81,82] that learn (optimize their internal parameters) progressively over time.

The neural network learning curves in this study were a good fit because both curves had fewer gaps between train and validation points, as shown in Appendix 3 for 2023. MOLUSCE-CA is a significant modeling approach as one of the artificial intelligence methods. CA-ANN algorithms have effectively assessed historical LULC and predicted the future trends of land use in BMNP.

### 3.7. Projection of LULC

**Fig 9:**
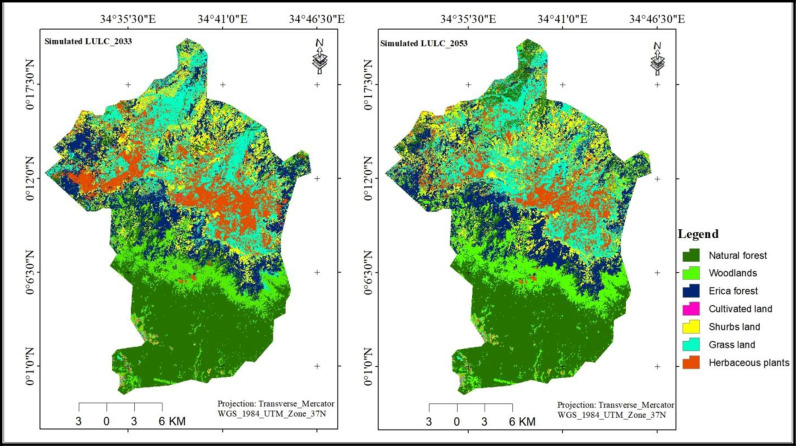
Projected LULC of BMNP (2033 and 2053).

After achieving satisfactory results from model validation, the land use and land cover (LULC) maps for 2033 and 2053 were projected using the CA-ANN model. The LULC maps for these years were simulated with additional iterations, and the results are illustrated in (Fig 9 and Table 11).

**Table 11:** Predicted areas of LULC classes and their percentage of change in BMNP (2023–2053).

LULC Type	LULC Area						% changes in area		
	2023		2033		2053		(2023-2033)	(2033-2053)	(2023-2053)
	(km^2^)	%	(km^2^)	%	(km^2^)	%			
Cultivated land	11.78	0.48	9.99	0.41	8.42	0.3	−15.17	−15.74	−28.52
Erica forest	297.87	12.24	293.61	12.06	288.09	11.8	−1.43	−1.88	−3.28
Grassland	409.41	16.82	398.44	16.37	331.51	13.6	−2.68	−16.80	−19.03
Herbaceous plants	205.08	8.43	211.90	8.71	215.53	8.9	3.32	1.71	5.10
Natural forest	704.37	28.94	668.66	27.47	658.23	27.0	−5.07	−1.56	−6.55
Shrubs land	483.86	19.88	508.98	20.91	559.22	23.0	5.19	9.87	15.57
Woodland	321.74	13.22	342.53	14.07	373.11	15.3	6.46	8.93	15.97

The future projections of LULC change indicate that, natural forest areas will remain the predominant land cover during this transition; however, its area is expected to decline by approximately 5.07%, 1.56%, and 6.55% from 2023 to 2033, 2033–2053, and 2023–2053, respectively.

While most LULC classes are projected to decrease by 2033 and 2053, significant reductions are anticipated in cultivated land, Erica forest, grassland, and natural forest areas. Specifically, these areas are expected to decline by approximately 28.51%, 3.28%, 19.03%, and 6.55%, respectively, between 2023 and 2053. The decrease in cultivated land may attribute to increased government efforts to protect the areas from human disturbance through community awareness initiatives. On the other hand, the reductions in Erica Forest, grasslands, and natural forests are probably driven by a combination of human activities and climate-related influences such as forest fires and the conversion of these areas to different land use types, as certain lower-altitude species shift to higher elevations due to climate warming. These were also mentioned by [24,25,83,84] as driving factors for changing LULC dynamic of the region.

In contrast, areas covered by shrubs, woodlands, and herbaceous plants are projected to increase by 46.58%, 26.84%, and 5%, respectively, between 2023 and 2053. This growth may be attributed to the expansion of suitable environments for these land use classes as they shift from lower to higher altitudes due to climate warming. The decline in areas of various land use types, such as grasslands, Erica forests, and natural forests in BMNP over the next three decades is likely to have adverse effects on the region’s biodiversity in general and the endemic and endangered Ethiopian wolf in particular [24,29,85]. The grasses in afro-alpine areas support common and giant mole rats, which are key components of the wolf’s diet.

## 4. Conclusion and recommendation

The objective of this study was to assess the land use and land cover (LULC) dynamics of BMNP, southeastern Ethiopia, from 1993 to 2023 and to the changes that might occur between 2033 and 2053Landsat 5 TM (1993), Landsat 7 ETM+ (2003), and Landsat 8 OLI-TIRS (2013 and 2023) data were obtained from the Google Earth Engine and classified using Random Forest (RF) algorithm while a CA-ANN was employed for modeling future LULC changes.

The analysis of LULC changes over the last 30 years (1993–2023) in the study area revealed a significant shift in land use patterns. Notably, areas of shrub land and woodlands have expanded, while cultivated land, Erica Forest, and grassland have decreased. Natural forests consistently remained the most dominant LULC type in BMNP, comprising approximately 31% to 33% of the total area. The LULC transition matrices highlight that woodlands and shrubs exhibited high instability, with significant portions converted into other land uses. In contrast, natural forests and cultivated lands were more stable LULC classes during the study period. The findings also revealed an increase in areas of shrubs land and woodlands along all elevation gradients.Althogh grassland and natural forest increased at lower altitudes, they sharply declined at the highest altitudes.

The LULC projections for 2033 and 2053 suggest that while natural forests will remain the primary land cover type, they are expected to experience a significant decline in the area over the next three decades. This highlights the need for conservation efforts to mitigate further loss of the forested regions. The results also indicate a complex shift in the landscape dynamics in 2053. While significant decreases are anticipated in cultivated land, Erica Forest, grassland, and natural forests, there is a notable increase in areas covered by shrubs, woodlands, and herbaceous plants are expected to increase by about 15.57%, 15.97% and 5% respectively. The out puts of this study can assist the conservation efforts by identifying priority areas that needs special attention based on ecological significance and threats.The methodologies employed in this study, particularly the use of Google Earth Engine and CA-ANN models, can also serve as a framework for other researchers studying similar ecosystems or issues.

The current and anticipated changes in land use and land cover (LULC) in the region placed an immense pressure on the unique biodiversity of the area, particularly the endemic and endangered Ethiopian wolf, a species emblematic of the region’s rich natural heritage. Addressing these issues necessitates collective action. Therefore, implementing community-based natural resource conservation and management practices are crucial in this area. To mitigate the rapid encroachments of woodlands and shrubs at the expense of grasslands, controlled and partial grazing practices are recommended for the afroalpine area.

## Supporting information

S1 Table Areas of LULC along elevation gradient in BMNP (1993–2023). (DOCX)

S2 Table Mean values and decadal differences of precipitation, maximum, and minimum temperatures of BMNP and across altitudinal gradients (1993–2023). (DOCX)

S3 Table Correlation of spatial variables used for LULC modeling. (DOCX)

S1 Fig Neural Network learning curve for transition potential modeling. (DOCX)

S2 Fig Model validation results. (DOCX)

S1 File Other supplementary materials. (DOCX)
