# Supplementary figures and images for: Modeling land use and land cover dynamics of Bale Mountains National Park using Google Earth Engine and cellular automata–artificial neural network (CA-ANN) model
Source: PLoS One. 2025 Apr 30;20(4):e0320428. doi: 10.1371/journal.pone.0320428 (PMC12043153; doi:10.1371/journal.pone.0320428)

**S1 Figure 2**: Model validation results


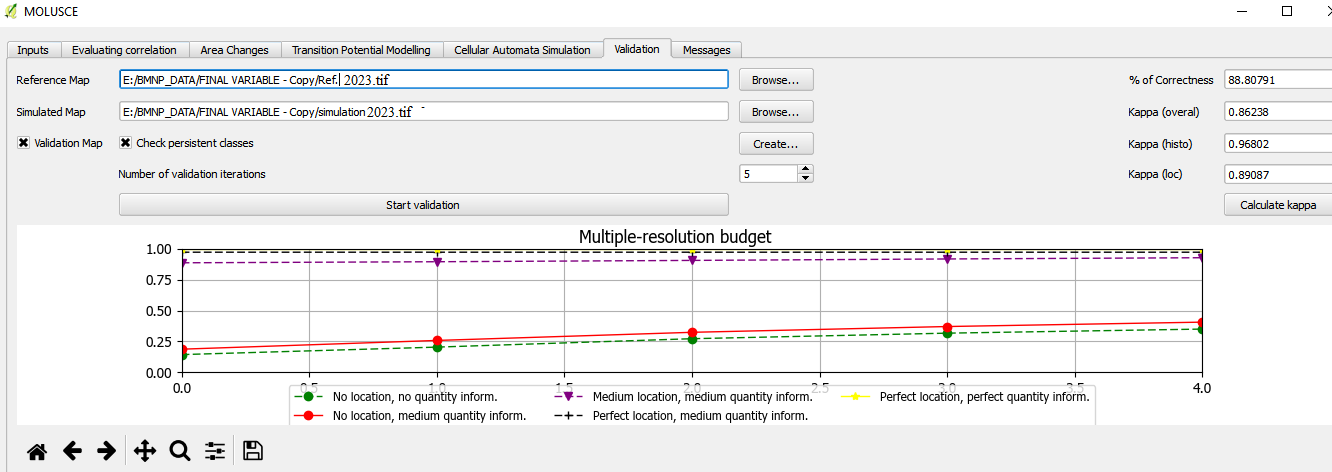

Supplement: S2 Table — (DOCX) [file pone.0320428.s002.docx]

**S1 Figure 1:** Neural Network learning curve for transition potential modeling


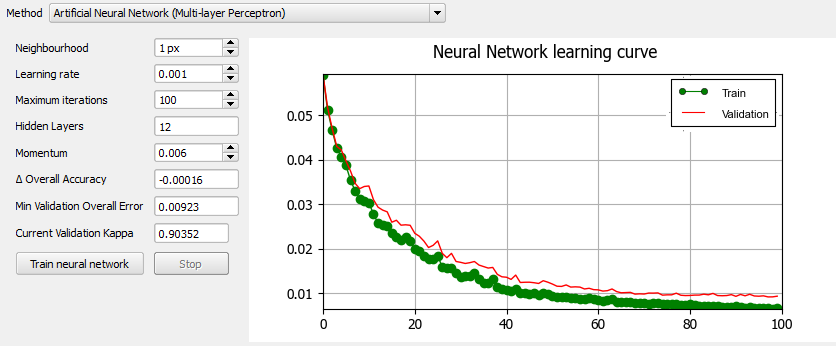

Supplement: S1 Fig — (DOCX) [file pone.0320428.s004.docx]
