# Supplementary material for: Modeling land use and land cover dynamics of Bale Mountains National Park using Google Earth Engine and cellular automata–artificial neural network (CA-ANN) model
Source: PLoS One. 2025 Apr 30;20(4):e0320428. doi: 10.1371/journal.pone.0320428 (PMC12043153; doi:10.1371/journal.pone.0320428)
Supplement: S2 Fig — (DOCX) [file pone.0320428.s005.docx]

S1 Table 3: Correlation of spatial variables used for LULC modeling

| **Spatial variables** | **correlation value** |
| --- | --- |
| Elevation  Minimum temperature  Maximum temperature  Precipitation | 0.86  0.79  0.73  0.48 |
| Distance from roads | -0.61 |
| Distance from towns  Slope | -0.64  -0.45 |
| Distance from streams | 0.01 |
| Population density | -0.02 |
