# Supplementary material for: Modeling land use and land cover dynamics of Bale Mountains National Park using Google Earth Engine and cellular automata–artificial neural network (CA-ANN) model
Source: PLoS One. 2025 Apr 30;20(4):e0320428. doi: 10.1371/journal.pone.0320428 (PMC12043153; doi:10.1371/journal.pone.0320428)

S1 Table 1: Areas of LULC along elevation gradient in BMNP (1993-2023)

|  |  | Area(sq.KM) | | |
| --- | --- | --- | --- | --- |
| **LULC Classes** | **Year** | <2600 | 2600-3200 | >3200 |
| **Natural forest** | 1993 | 653.1 | 102.0 | 5.4 |
|  | 2003 | 693.5 | 98.7 | 7.0 |
|  | 2012 | 653.9 | 104.6 | 5.7 |
|  | 2023 | 673.8 | 28.7 | 1.8 |
| **Woodlands** | 1993 | 179.7 | 35.9 | 19.8 |
|  | 2003 | 153.6 | 45.8 | 13.8 |
|  | 2012 | 160.7 | 69.3 | 27.3 |
|  | 2023 | 210.2 | 61.2 | 50.4 |
| **Erica forest** | 1993 | 41.8 | 316.8 | 39.1 |
|  | 2003 | 35.8 | 309.6 | 43.7 |
|  | 2012 | 28.3 | 235.6 | 47.9 |
|  | 2023 | 19.9 | 225.8 | 52.2 |
| **Shrubs land** | 1993 | 18.9 | 42.3 | 197.3 |
|  | 2003 | 29.0 | 38.5 | 234.0 |
|  | 2012 | 5.2 | 63.0 | 345.1 |
|  | 2023 | 23.3 | 51.5 | 409.0 |
| **Grass land** | 1993 | 22.7 | 29.2 | 474.3 |
|  | 2003 | 29.6 | 27.9 | 410.5 |
|  | 2012 | 27.6 | 37.4 | 389.3 |
|  | 2023 | 30.5 | 38.6 | 340.3 |
| **Herbaceous plants** | 1993 | 1.8 | 14.1 | 219.7 |
|  | 2003 | 0.9 | 12.0 | 227.1 |
|  | 2012 | 3.2 | 14.2 | 203.7 |
|  | 2023 | 0.3 | 3.2 | 201.5 |
| **Cultivated land** | 1993 | 5.1 | 1.2 | 13.8 |
|  | 2003 | 0.0 | 0.6 | 22.4 |
|  | 2012 | 0.5 | 1.5 | 10.0 |
|  | 2023 | 1.7 | 1.0 | 9.1 |

S1 Table 2: Mean values and decadal differences of precipitation, maximum, and minimum temperatures of BMNP and across altitudinal gradients (1993-2023)

| Altitude | Years | Altitudes(m.a.s.l) | | | | | |
| --- | --- | --- | --- | --- | --- | --- | --- |
|  |  | < 2000 | 2000-2600 | 2600-3200 | 3200-3800 | > 3800 | Mean |
| Precipitation | 1990-1999 | 1000.4 | 1010.9 | 1054.7 | 1039.4 | 1027.1 | 1026.5 |
|  | 2000-2009 | 954.0 | 964.1 | 1004.5 | 975.3 | 969.0 | 973.4 |
|  | 2010-2019 | 921.6 | 927.9 | 944.3 | 916.6 | 925.0 | 927.1 |
|  | Difference | -78.7 | -83.0 | -110.4 | -122.8 | -102.1 | -99.4 |
| Maximum temperature | 1990-1999 | 17.7 | 17.3 | 17.9 | 18.6 | 16.8 | 17.7 |
|  | 2000-2009 | 18.7 | 18.4 | 18.9 | 19.6 | 18.0 | 18.7 |
|  | 2010-2019 | 19.3 | 19.0 | 19.4 | 20.1 | 18.7 | 19.3 |
|  | Difference | 1.6 | 1.7 | 1.4 | 1.5 | 1.9 | 1.6 |
| Minimum temperature | 1990-1999 | 3.9 | 3.7 | 4.1 | 4.5 | 3.5 | 3.9 |
|  | 2000-2009 | 5.3 | 5.1 | 5.3 | 5.8 | 4.8 | 5.2 |
|  | 2010-2019 | 5.7 | 5.5 | 5.7 | 6.2 | 5.3 | 5.7 |
|  | Difference | 1.7 | 1.8 | 1.7 | 1.7 | 1.8 | 1.7 |

S1 Table 3: Correlation of spatial variables used for LULC modeling

| **Spatial variables** | **correlation value** |
| --- | --- |
| Elevation  Minimum temperature  Maximum temperature  Precipitation | 0.86  0.79  0.73  0.48 |
| Distance from roads | -0.61 |
| Distance from towns  Slope | -0.64  -0.45 |
| Distance from streams | 0.01 |
| Population density | -0.02 |

**S1 Figure 1:** Neural Network learning curve for transition potential modeling


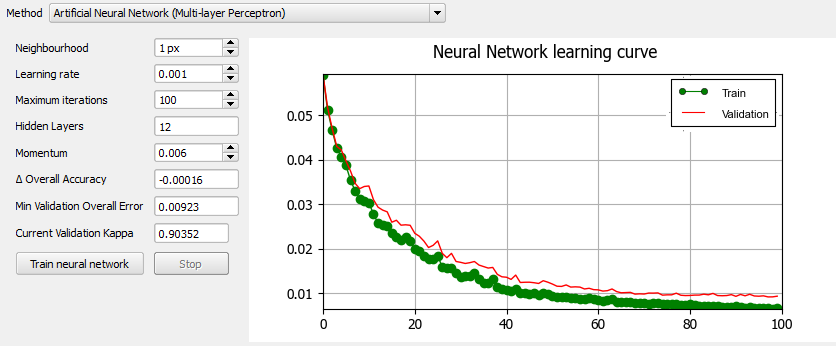


**S1 Figure 2**: Model validation results


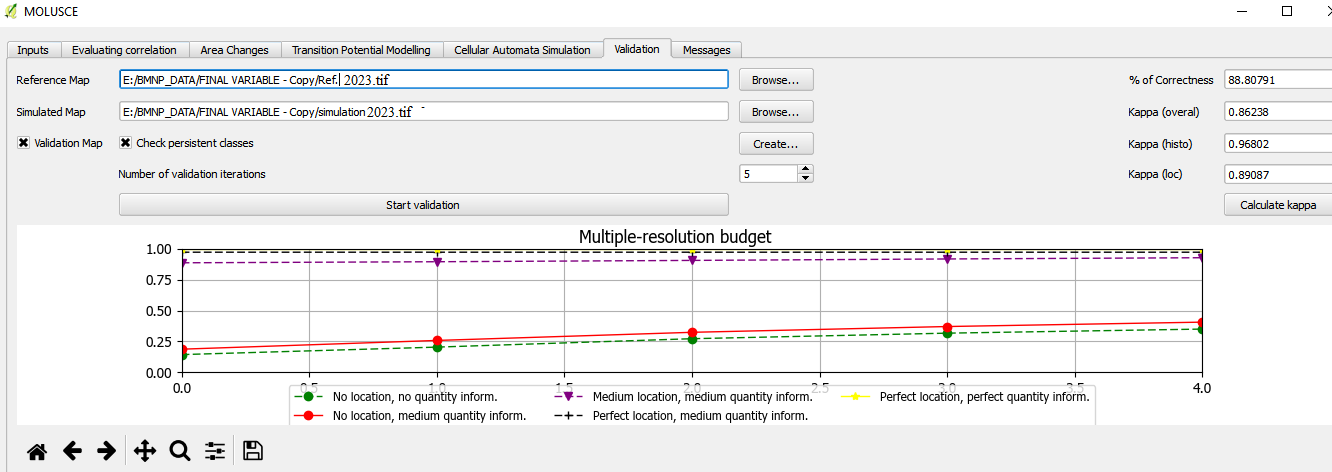

Supplement: S1 File — (DOCX) [file pone.0320428.s006.docx]
